# Preparation of magnetic microalgae composites for heavy metal ions removal from water

**DOI:** 10.1016/j.heliyon.2024.e37445

**Published:** 2024-09-05

**Authors:** Huan-Cheng Lin, Yi-Ju Liu, Da-Jeng Yao

**Affiliations:** a Department of Power Mechanical Engineering, National Tsing Huiversity, Hsinchu, Taiwan; b Food Industry Research and Development Institute, Hsinchu, Taiwan; c Institute of NanoEngineering and MicroSystems, National Tsing Hua University, Taiwan

**Keywords:** Microalgae, Magnetic nanoparticle, Removal of metal ion

## Abstract

Hexavalent chromium Cr(VI) and divalent Copper Cu(II) ions were heavy metals that were severely toxic to organisms and aquatic ecosystems. Algae is considered as an eco-friendly and cost-effective method for heavy metal ions treatment, but there are still some disadvantages to be improved. Therefore, In this paper, we combine microalgae biomass with ferric oxide magnetic nanoparticles (MNPs) to prepare a more widely applicable adsorbent. Box–Behnken design (BBD) was evaluated for exploring the significant parameters for maximum adsorption in a binary Cr(VI) and Cu(II) solution using our synthesized MNPs@Algae (M@A) adsorbent and constructed a predictability of 88.84 and 95.6 % quadratic regression model, through ANOVA, Pareto Chart of the standardized effects, Three-dimensional surface plot, desirability function to analysis and discussion each factor further. The combined results from UV–Vis, FTIR, TGA, and SQUID measurements confirmed the successful synthesis and accurate properties of the MNPs@Algae composites. The experiment results indicated that when initial pH 6, 5 mg/L Cr(VI), 20 mg/L Cu(II), M@A(3 : 3), dose (1 g/L), and contact time 6 h can achieve the maximum 58 % Cr(VI) and 73.4 % Cu(II) removal efficiency. M@A can eliminate Cr(VI) and Cu(II) from binary solution and separate them from the solution within a few seconds by a permanent magnet as a feasible and efficient absorbent.

## 1 Introduction

In recent decades, with the intensive development of industrial technology and increased of human population, various industries have directly or indirectly discharged polluted liquid wastes into rivers or oceans, which will have adverse effects on the entire ecosystem [1]. Among them, the pollution of heavy metals is the most concerned, due to its high toxicity, non-biodegradability, and bioaccumulation, even if the concentration released into the environment is not high, it will pose serious health risks to the human body and other organisms. Accumulation in the food chain, thereby aggravating the deterioration of ecological quality [2] (see Fig. 9).

Chromium has been identified as one of the most dangerous toxic metals. In industry, it is widely used in metal processing, electroplating, and leather manufacturing. In addition, chromate is often used to prevent the corrosion of equipment by circulating water in the production process. Chromium mainly exists in soil and water in the form of Cr(III) and Cr(VI) ions, and Cr(VI) is usually far more toxic than Cr(III), has a high solubility in water, and is easily absorbed and poisoned into the human body. Accumulation in the body, like kidney, stomach, and liver, would causes carcinogenic [3]. Copper is the earliest metal used by human beings and one of the indispensable elements for human health. It is used in a high amount in industry and agriculture. The World Health Organization (WHO) [4] and the United States Environmental Protection Agency (EPA) [5] have set permissible limits for various heavy metals in drinking water to mitigate these risks. For example, the permissible limit for Cr(VI) is 0.05 mg/L and for Cu(II) is 1.3 mg/L. Therefore, the possibility of copper pollution is also relatively high. It is relatively less toxic by itself, but excessive intake will also cause kidney damage, liver disease, anemia, and intestinal irritation [6].

So far, traditionally, many technologies have been developed to remove heavy metal ions from water through physical or chemical treatment, such as chemical precipitation [7], membrane filtration [8], adsorption [9], electrochemical treatment [10], ion exchange resin [11], and so on. However, although these technologies have certain advantages, most of them have some disadvantages. e.g. the residual volume and toxicity of sludge, high maintenance, energy consumption, and investment costs. Bio-sorbents derived from natural ecology have recently become the focus of attention due to their lower cost, higher efficiency, and more environmental friendliness. Bio-sorbents include agricultural wastes [12], plants [13], algae, fungi, and bacteria [14]. The polysaccharides, lipids, and proteins contained in the cell wall, are composed of various functional groups, and bind to various pollutants by actions including microprecipitation, chelation, complexation, ion exchange, and physical adsorption [15]. Among them, microalgae is considered as the potential absorbent material for the removal of heavy metals because of outstanding adsorption capacity, no toxic wastes, low cost, convenient handling, and biodiversity [16]. However, in the actual environment, requirements such as centrifugation or filtration to separate the microalgae from the aqueous phase, would consume a lot of time and cost, and limit to be used. To overcome this disadvantage, the magnetic nanoparticle can be used for more efficient absorbent.

In this research, microalgae with iron oxide magnetic nanoparticles, Fe_3_O_4_ MNPs, can form a composite adsorption material. The collection and separation can be carried out more quickly by using an external magnetic field. The chemical co-precipitation method was chosen for synthesizing magnetic nanoparticles (MNPs) due to its simplicity, high yield, cost-effectiveness, and environmental friendliness. This method does not require complex equipment or harsh conditions, making it suitable for large-scale production while minimizing environmental impact [17,18].

In addition, this study performs a binary Cr(VI) and Cu(II) solution as an adsorption environment and using Box–Behnken design (BBD) [19] to explore the effect of the independent variables in the adsorption process and the competitive adsorption of Cr(VI) and Cu(II).

## 2 Material and methods

### 2.1 Chemicals, reagents, and solutions

The stock of 1000 mg/L Cr(VI) and Cu(II) solution were prepared by dissolving the exact quantities of K_2_CrO_4_ and CuSO_4_·5H_2_O in deionized water, respectively. 2.5 g 1, 5-diphenylcarbazide (DPC, 98 %, ALFA, U.K) was dissolved in 50 mL acetone and 0.1 % Sodium diethyldithiocarbamate trihydrates (Na· DDTC, ALFA, U.K) as developer for Cr(VI) and Cu(II) respectively [20]. The desired initial pH value of each solution was adjusted by 0.1 M NaOH and 0.1 M H_2_SO_4_.

### 2.2 Cultivation and preparation of bio-sorbent

The microalgae, chlorella sp., was purchased from the website of Bioresource Collection and Research Center (BCRC). All *Chlorella* sp. were grown on culture medium (0.15 g/L Ca(NO_3_)_2_·4H_2_O, 0.1 g/L KNO_3_, 0.05 g/L β–Na_2_glycerophosphate·5H_2_O, 0.04 g/L MgSO_4_·7H_2_O, 0.1 μg/L Vitamin B12, 0.1 μg/L Biotin, 10 μg/L Thiamine HCl, 0.5 g/L Tris (hydroxymethyl) aminomethane, 3 mL/L PIV metals(1 g/L Na_2_EDTA·2H_2_O, 0.196 g/L FeCl_3_·6H_2_O, 0.036 g/L MnCl_2_·4H_2_O, 0.0104 g/L ZnCl_2_, 0.004 g/L CoCl_2_·6H_2_O, 0.0025 g/L Na_2_MoO_4_·2H_2_O), and 8.82 mg/L sodium citrate agar (1.5 % w/v) plates. All *Chlorella* sp. were cultured in culture medium containing 50 mM NaHCO_3_ in 500 mL shake flasks. Cultures were maintained in a photoperiod of 12:12 h at a light intensity of 60 μmol photons/m^2^sec at 25 °C. After reaching the stationary phase, cells were harvested by centrifugation (4200 rpm for 8 min), washed twice with deionized water, dried in an oven at 55 °C for 24 h, ground into fine particle and stored in a fridge.

### 2.3 Preparation of Fe_3_O_4_ MNPs

In the synthesis of Fe_3_O_4_ magnetic nanoparticles (MNPs), using the chemical co-precipitation method, according to the ratio of Fe(II): Fe(III) to be 1 : 2, it is prepared in an oxygen-free nitrogen environment. About 3.1 g FeCl_3_·6H_2_O and 2.1 g的FeSO_4_·7H_2_O was adding to 80 mL deionized water [21], and 0.1 M NaOH was slowly added dropwise until pH to be 9, then reaction for 90 min at 55 °C with a magnetic stirring bar. The particles were cooled down to room temperature, separated by magnet, and washed with deionized water and ethanol to remove unreacted chemicals. The collected MNPs were dried in an oven at 55 °C and storage at 4 °C for further applications.

### 2.4 Preparation of Fe_3_O_4_ MNPs@Algae (M@A)

Three different ratios, 3:1, 3:3, and 3:5, of M@A adsorbent were prepared by mixing different weight percentages of chlorella. biomass. To obtain 0.3 g of Fe_3_O_4_ MNPs, 0.465 g of FeCl_3_·6H_2_O and 0.315 g of FeSO_4_·7H_2_O were dissolved in 80 mL of deionized water, and dropwise NaOH (0.1 M) until pH 9 slowly during stirring. Take three different weight, 0.1 g, 0.3 g, and 0.5 g, of ground dried biomass and redissolve with an ultrasonic cleaner for 20 min. Mix above solution at 55 °C and stirred for 90 min under a nitrogen atmosphere. After cooling, the particles were washed with deionized water and ethanol three times with the assistance of magnetic decantation and dried in an oven at 55 °C, then stored for further applications.

### 2.5 Batch adsorption analysis by Box-Behnken design (BBD)

Response surface methodology (RSM) is a method developed by combining mathematics and statistics, which is used to explore the mathematical model relationship among more than three independent variables and the response function. In the presented study, the most widely Box-behnken design (BBD) was used, and the design concept is shown in Table 1. The experimental design software Design Expert 13 generates 3 center points and 27 different experimental designs as four independent variables on the simultaneous adsorption of Cr(VI) and Cu(II) were investigated. These four variables will be evaluated by setting three codes (−1, 0, +1) at equal intervals, including pH (A: 2, 4, 6), ratio of M@A (B: 3:1, 3:3, 3:5), Cr(VI) concentration (C: 2.5, 5, 7.5 mg/L) and adsorbent dosage (D: 0.5, 1.0, 1.5 g/L). Then using Eq. (1) represents the correlation among the variable and a function of the response (removal efficiency of Cr(VI) and Cu(II)). All experiments were performed at a fixed Cu(II) concentration of 20 mg/L with continuous stirring and mixing with a magnetic stirrer for 6 h at room temperature.

(1) $$Y=b_{0}+b_{1}A+b_{2}B+b_{3}C+b_{4}D+b_{11}A^{2}+b_{22}B^{2}+b_{33}C^{2}+b_{44}D^{2}++b_{12}AB+b_{13}AC+b_{14}AD+b_{23}BC+b_{24}BD+b_{34}CD+\varepsilon$$

in which Y is the predicted Cr(VI) or Cu(II) removal efficiency (%); A, B, C, D is the coding level of the selected variable; $b_{0}$ is the regression coefficient; $b_{1},{\;b}_{2},{\;b}_{3},{\;b}_{4}$ are the linear coefficients; $b_{11},{\;b}_{22},{\;b}_{33},{\;b}_{44}$ are the quadratic coefficients; $b_{12},{\;b}_{13},{\;b}_{14},b_{23}\text{,}$ $b_{24},b_{34}$ are the interaction coefficients; and ε is the residual term.

**Table 1:** Variables and levels in BBD.

Variable	Code and actual levels			
	Variable code	Low (−1)	Central (0)	High (+1)
pH	A	2	4	6
M@A Ratio	B	3:1	3:3	3:5
Cr(VI) Conc. (mg/L)	C	2.5	5	7.5
Dose (g/L)	D	0.5	1	1.5

### 2.6 Cr(VI) and Cu(II) analysis method

After adsorption experiments, the adsorbent was separated by a permanent magnet. Then, using UV/visible spectrophotometer (JASCO, model V-670) to analyze separated supernatant. by adding developer (DPC or DDTC) to the samples, a calibration line between absorbance and standard Cr(VI) or Cu(II) concentrations was established at the wavelength of 540 nm and 450 nm, respectively [22]. According to quantitative analysis by determining the concentration, the removal efficiency (%) was determined by Eq. (2), and adsorption capacity ($q_{e}$, mg/g) was estimated by Eq. (3).

(2) $$Removal\left(\%\right)=\frac{{C_{0}-C}_{e}}{C_{0}}\times 100\%$$

(3) $$q_{e}\left(mg/g\right)=\frac{\left({C_{0}-C}_{e}\right)V}{M}$$

in the above equations, C_0_ and C_e_ (mg/L) represent the concentration of the solution at the initial and equilibrium states, respectively; *q*_*e*_ indicate the adsorption amount per unit of adsorbent (mg/g); V is the volume of the solution in Liter; M is the adsorbent weight in gram.

### 2.7 Desorption and reuse procedure

**Fig. 1:**
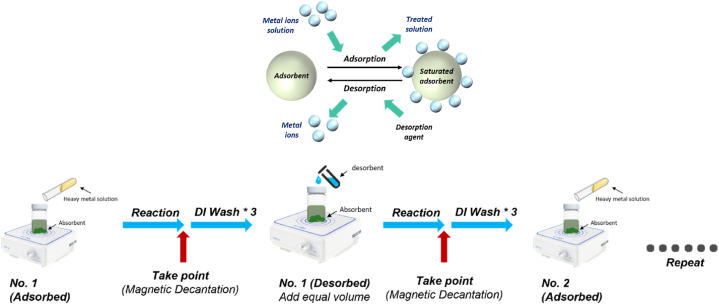
The illustration of the mechanism and operation flow of the adsorption-desorption cycle.

To reduce the processing cost which can improve the usability of further application, M@A adsorbent material can be reused to maintain its adsorption capacity after multiple adsorption-desorption cycles. In the desorption experiment, 0.5 M NaOH is added with 2.5 M NaCl and 0.2 M HCl, as the desorption regent of Cr(VI) and Cu(II), respectively [23], the reaction was continuously stirred at room temperature. The desorption efficiency of the adsorbent was calculated in Eq. (4) by analyzing the metal ion concentration of the supernatant. The operation steps are shown in Fig. 1:

(4) $$Desorption\;efficiency\left(\%\right)=\frac{Amount\;of\;metal\;ions\;desorbed}{Amount\;of\;metal\;ions\;adsorbed}\times 100\%$$

## 3 Results and discussion

### 3.1 Preliminary study

**Fig. 2:**
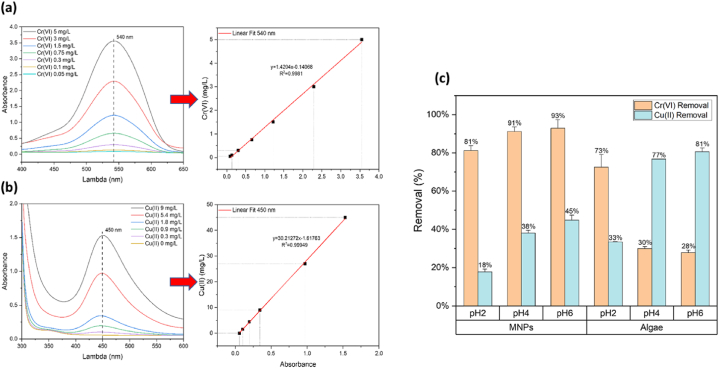
(a) Establish calibration line in 540 nm by absorbance with different standard Cr(VI) conc.; (b) establish calibration line in 450 nm by absorbance with different standard Cu(II) conc.; (c) Result of MNPs and algae under a binary Cr(VI) and Cu(II) solution (Cr(VI) 1.75 mg/L, Cu(II) 7.5 mg/L, dose 1 g/L, contact time 3 h).

Establish a binary Cr(VI) and Cu(II) solution using as stock standard solution, and in quantitative analysis of UV–vis, 20 μL of DPC was added to 1 mL of supernatant, adjusted to pH 2, then calculating back Cr(VI) concentration with calibration line through the absorbance at 540 nm; 0.2 mL of DDTC. 0.2 mL of supernatant were added to 0.6 mL of DI water, adjusted to pH 8, and calculating back Cu(II) concentration with calibration line through the absorbance at 450 nm, as shown in Fig. 2.

First, we explored the adsorption capacity of MNPs and chlorella biomass in different pH values (2, 4, 6) for binary Cr(VI) and Cu(II) solution. Fig. 2c shows that either MNPs or microalgae are affected the removal of metal ions with different pH environment, and fail to have a suitable condition for simultaneous adsorption by mixing them together. For example, at pH 6, MNPs achieved a 93 % removal rate for Cr(VI) but only 45 % for Cu(II). In contrast, at the same pH, *Chlorella* biomass showed lower efficiency in removing Cr(VI) (28 %) compared to Cu(II) (81 %). This significant difference in removal rates at various pH levels emphasizes the challenge in finding an optimal mixed condition that effectively adsorbs both metal ions. The adsorption mechanism is primarily driven by the biological material. Functional groups such as carboxyl, amino, and hydroxyl groups on the algae biomass facilitate the binding of heavy metal ions through complexation, ion exchange, and electrostatic interactions [24,25]. Specifically, carboxyl groups can donate lone pairs of electrons to form complexes with metal ions, amino groups can participate in ion exchange due to their positive charge under certain pH conditions, and hydroxyl groups can contribute to electrostatic interactions. Studies have shown that these functional groups are crucial for the biosorption process, as they provide specific sites for metal ion binding [26,27]. Recent research has focused on developing efficient adsorbent materials for removing hexavalent chromium (Cr(VI)) and other metal ions from industrial wastewater. For example, a core-shell/bead-like alginate@polyethyleneimine (PEI) material demonstrated excellent adsorption capacity and regeneration performance for Cr(VI) [17]. Alginate hydrogels with a multi-cavity structure, modified with PEI, showed high-efficiency Cr(VI) removal [18]. Hollow PEI/carboxymethyl cellulose (PEI/CMC) beads efficiently removed Cr(VI) and phosphates [19]. A bi-layered hollow amphoteric composite also displayed superior performance in removing multiple metal ions [20]. These studies highlight the importance of material design and modification in enhancing adsorption performance.

The magnetic nanoparticles complement this process by providing a large surface area for adsorption and facilitating easy recovery and separation through an external magnetic field. Their surface can be functionalized to enhance interaction with the biological material, making the combined system more effective [28]. The magnetic nanoparticles mainly provide a means for easy recovery and separation through an external magnetic field. Therefore, we expect that mix MNPs and *Chlorella* biomass in different ratios, combining them to achieve better dual functions of adsorption and magnetic separation.

### 3.2 Characterizations of adsorbents

**Fig. 3:**
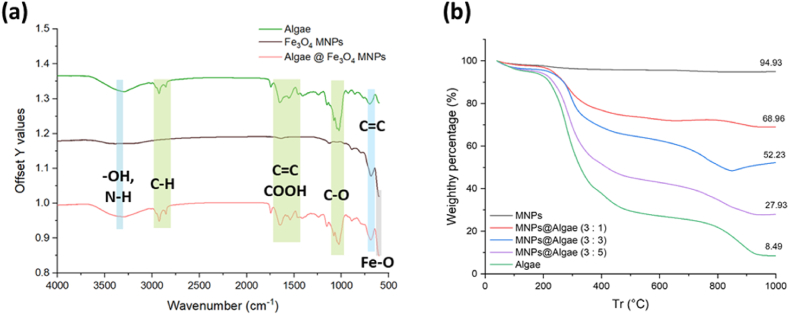
Result of adsorbent characterizations. (a) by FTIR; (b) by TGA.

The FT-IR spectra of algae biomass, Fe_3_O_4_ MNPs and M@A (1 : 1) are shown in Fig. 3a. The results show that there are many characteristic functional groups on the surface of microalgae biomass, mainly including C=C(665 - 730 cm^−1^), C-O (1087 - 1124 cm^−1^), COOH (1395 - 1440 cm^−1^), C=C (1735 - 1750 cm^−1^), C-H (2695 - 2830 cm^−1^), O-H (3200 - 3600 cm^−1^). Fe_3_O_4_ MNPs show that the main peak is Fe-O (590 cm^−1^) [29], and the rest have no other characteristic functional groups except for the weak O-H (3200 - 3600 cm^−1^). When Fe_3_O_4_ MNPs and algae biomass are combined, the FT-IR spectra of M@A (1:1) show the presence of these characteristic peaks with almost unchanged intensities. This indicates that the binding between algae biomass and Fe_3_O_4_ MNPs is successful. Specifically, the persistence of the characteristic peaks of algae biomass (such as C-O, COOH, C-H, and O-H) suggests that these functional groups are involved in the binding process. Additionally, the presence of the Fe-O peak confirms that Fe_3_O_4_ MNPs maintain their structural characteristics during the combination process. Therefore, the observed peaks and their intensities in the FT-IR spectra of M@A (1:1) provide evidence of the successful binding between algae and Fe_3_O_4_ MNPs. In other studies, it has also been observed that when biomaterials are combined with Fe_3_O_4_ MNPs, the peak Fe-O bond can still be observed [30,31].

TGA analysis was used to explore the composition percentages of the prepared M@A. The detection results of TGA are shown in Fig. 3b. Microalgae biomass is mainly divided into three stages during thermal cracking [32], (1) 30–200 °C, most of the moisture and highly volatile compounds are lost at this time, (2) 200–550 °C, the main organic compounds of the microalgal biomass (e.g. lipids, proteins, and carbohydrates) both are pyrolyzed at this point, also known as the active pyrolytic zone, which is the area where the most biomass is lost, (3) 550–800 °C, where thermally stable compounds decompose and form Biochar loses about 91.5 % of the total weight; Fe_3_O_4_ MNPs loses about 5 % of the total weight until it burns to 1000 °C; M@A are prepared by mixing with different weight percentages, and the results show that the combustion reaches 1000 °C, the total weight loss was about 31 %, 48 %, and 72 %, respectively. TGA analysis showed a mass loss rate of 94.93 % of MNPs, indicating high thermal stability. From the above results, it can be speculated that the residual biochar and most of the magnetic particles formed by microalgae at this time also indicated that the prepared adsorbent contained algae and Fe_3_O_4_ MNPs weight percentages are still roughly the same.

**Fig. 4:**
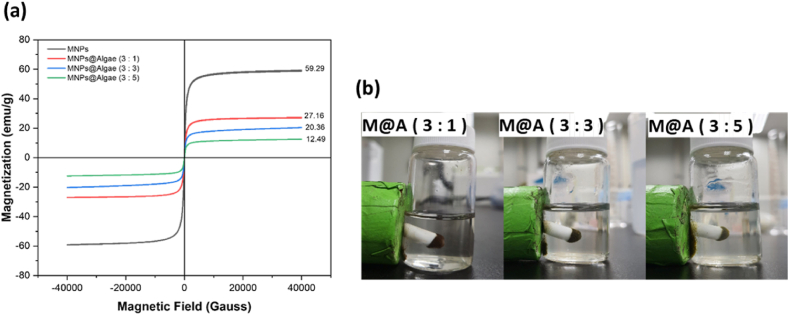
Results of magnetic properties. (a) by SQUID, (b) separation by a permanent magnet.

Magnetic properties were investigated at 300 K by using SQUID. The results, Fig. 4a, show that all magnetic adsorbents have no obvious hysteresis under the change of magnetic field. The saturation magnetization of Fe_3_O_4_ MNPs is about 60 emu/g. Pure MNPs samples have the highest magnetization (59.29 emu/g). As the proportion of algae increases, the magnetization significantly decreases, with MNPs@Algae (3:1) at 27.16 emu/g, MNPs@Algae (3:3) at 20.36 emu/g, and MNPs@Algae (3:5) at 12.49 emu/g. This indicates that the introduction of algae dilutes the magnetism of MNPs, and as the proportion of algae increases, the magnetism weakens. Under different ratios of M@A, the saturation magnetization also decreases gradually with the increase of the proportion of mixed microalgae. These data demonstrate the magnetic properties of MNPs and their changes when combined with algae, indicating that the magnetism of the composite material can be regulated by adjusting the ratio of MNPs to algae. In addition, Fig. 4b demonstrates that the different ratios of M@A in an aqueous solution can be readily separated by a permanent magnet in a few seconds, which can greatly reduce the cost of separating the adsorbent time.

### 3.3 Statistics analysis of binary adsorption of Cr(VI) and Cu(II) by M@A

In the current study, different ratios of M@A were used as adsorbents to conduct adsorption experiments on aqueous solutions with different Cr(VI) concentrations and fixed Cu(II) concentrations concurrently. Use Box-Behnken Design (BBD) to clearly study the degree of influence of various factors on the adsorption process, and fit the experimental results with a mathematical model to predict the optimal adsorption conditions and maximize the removal percentage. In the experiment, a total of 27 experiments were carried out [33], and the four independent variables included: pH (A), mixing ratio of M@A (B), Cr (VI) concentration (C) and adsorbent dose (D), while the response values were expressed as the removal percentages of Cr(VI) and Cu(II), respectively.

Table 2 presents the actual factor levels of the four independent variables in the BBD, the experimental removal values of Cr(VI) and Cu(II), and predicted values. The results showed that the removal values of Cr(VI) ranged from 27.9 to 98.8 %, and the removal values of Cu(II) ranged from 13.2 to 75.1 %. The maximum removal percentages of both Cr(VI) and Cu(II) were obtained in the no.12 experiment with a percent of 58 % for Cr(VI) and 73.4 % for Cu(II) while at pH 6, M@A(3 : 3), 5 mg/L Cr(VI) concentration @ 1.5 g/L dose, culturing under 6 h.

**Table 2:** BBD matrix of four variables with actual factor levels, experimental and predicted values of adsorption Cr(VI) and Cu(II).

No.	pH	Ratio	Conc. Cr(VI) (mg/L)	Conc. Cu(II) (mg/L)	Dose	Exp. Cr(VI) (%)	Exp. Cu(II) (%)	Fit. Cr(VI) (%)	Fit. Cu(II) (%)
1	2	1	5	20	1	91.1	17.3	90	19.2
2	6	1	5	20	1	52.9	70.5	58	68.3
3	2	5	5	20	1	82.5	21.9	74	22.9
4	6	5	5	20	1	42.5	75.1	40.2	72
5	4	3	2.5	20	0.5	44.8	33.4	43.5	32.7
6	4	3	7.5	20	0.5	33.8	36.6	29.4	36.5
7	4	3	2.5	20	1.5	92	35.8	93	34.7
8	4	3	7.5	20	1.5	39.3	46.1	37.2	45.6
9	2	3	5	20	0.5	58.2	21	61.4	19.5
10	6	3	5	20	0.5	32.8	68.1	33.5	68.6
11	2	3	5	20	1.5	93.3	26.2	95	25.1
12	6	3	5	20	1.5	58	73.4	57.2	74.2
13	4	1	2.5	20	1	80.5	30.9	81.3	31.4
14	4	5	2.5	20	1	61	31	62.7	33.4
15	4	1	7.5	20	1	43.9	40.1	44.6	37.1
16	4	5	7.5	20	1	27.9	43.5	29.5	42.5
17	2	3	2.5	20	1	98.8	17.2	99.5	15.6
18	6	3	2.5	20	1	65.5	55.6	62.5	56.2
19	2	3	7.5	20	1	56.3	13.2	60.4	14.4
20	6	3	7.5	20	1	31.3	68.6	31.6	72
21	4	1	5	20	0.5	45.4	40.3	43	41.7
22	4	5	5	20	0.5	30	39.2	34	39.6
23	4	1	5	20	1.5	82.6	40.2	79.5	41.5
24	4	5	5	20	1.5	51.3	50.6	54.7	51
25	4	3	5	20	1	53.6	38.7	56.2	39.4
26	4	3	5	20	1	58.6	40.6	56.2	39.4
27	4	3	5	20	1	56.4	39	56.2	39.4

### 3.4 Three-dimensional surface plots for Cr(VI) and Cu(II) removal

**Fig. 5:**
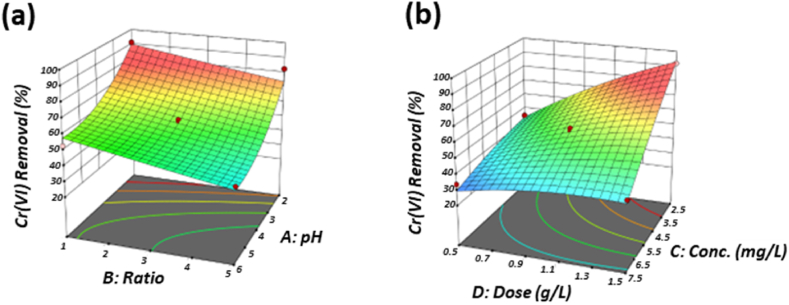
Three-dimensional surface plots for Cr(VI) removal. (a) pH-Ratio; (b) Cr(VI) concentration.-Dose.

Fig. 5a shows the effect of pH(A) and Ratio(B) on removal of Cr(VI), while fixed other variables at their central level (Cr(VI) concentration (C) and Dose(D)). The results show that, at lower pH and lower Ratio, the removal rate of Cr(VI) is relatively high. And with the decrease to lower pH value, the increase of the removal rate is also increased, while the ratio maintains a linear increase. Fig. 5b shows the effect of Cr(VI) concentration (C) and dose(D), as the dose was 0.5 g/L, the change of Cr(VI) concentration only had a slight effect on the removal rate of Cr(VI), but when the dose gradually increased, the effect of the initial Cr(VI) concentration became more obvious, which may be due to the saturation of active binding sites on the adsorbent surface.

**Fig. 6:**
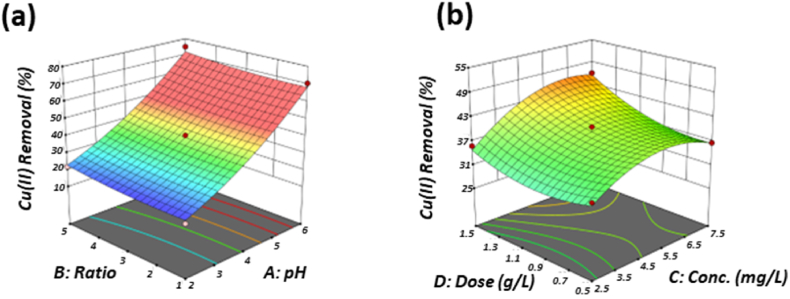
Three-dimensional surface plots for Cu(II) removal. (a) pH-Ratio; (b) Cr(VI) concentration -dose.

Similarly, presents the effect of the removal rate of Cu(II) at a fixed concentration at pH(A), Ratio(B), Cr(VI) concentration (C), and dose(D). Fig. 6a shows the effect of pH(A) and Ratio(B) on the fixed concentration of Cu(II), while other variables are under their central level. Obviously, the increase of pH value has a better removal rate of Cu(II), which is much greater than the change under the Ratio. In addition, Fig. 6b shows the effect of Cr(VI) concentration (C) and dose(D), it presented that the higher level of Cr(VI) concentration increases the percentage of Cu(II) removal by fixed dose, and the amount of adsorbent also increases the removal rate of Cu(II), but the influence of these two variables is still much smaller than the pH value.

### 3.5 Effect of initial pH value and the ratio of M@A

The pH value affects both the degree of ionization of metal ions and the surface characteristics of the adsorbent. Cr(VI) can exist in different forms at different pH values, namely HCrO_4_^−^, Cr_2_O_7_^−^, and CrO_4_^2−^. When the pH value is greater than 6, Cu(II) will form Cu(OH)_2_ with OH- and precipitate. In acidic aqueous solutions, Cr(VI) and copper mainly exist in the form of anionic HCrO_4_^−^ and cationic Cu(II), respectively. Therefore, when the pH value is less than 4, a large amount of H^+^ will occupy the surface functional groups of the algae in the adsorbent and be protonated, turning it into a positive charge, resulting in a greater electrostatic attraction with the anion HCrO_4_^−^. While for the cationic Cu(II), due to it needs to compete with H^+^ ions for binding sites and the repulsive force increases, while the adsorption capacity is greatly reduced. With the increase of pH value, the H^+^ in the solution is gradually converted into OH- and deprotonated, so it is more unfavorable for the removal of Cr(VI) and more favorable for the adsorption of Cu(II). Previous studies indicate that microalgae-montmorillonite composites are highly effective in adsorbing heavy metals, with the adsorption efficiency of Cr(VI) and Cu(II) significantly influenced by pH, achieving maximum capacities at specific pH levels [34]. Furthermore, the optimal pH conditions for maximum Cr(VI) and Cu(II) removal from chemically modified *Chlorella* and *Spirulina platensis* biomass were pH 2 and 6, respectively [35]. In our study showed that the algae combined with the Fe_3_O_4_ MNPs still have a certain ability to remove Cr(VI) at higher pH and maintain a good adsorption force for Cu(II), which also makes this adsorbent can be used in a wider range of conditions.

### 3.6 Effect of initial Cr(VI) concentration and dose

The change in initial Cr(VI) concentration and adsorbent dose corresponds to the relationship between adsorbate present in solution and available active sites. Increasing the initial Cr(VI) concentration will decrease the removal efficiency of Cr(VI), but increased the removal efficiency of Cu(II) under the fixed adsorbent, which may be due to the availability of available sites on the adsorbent surface are limited, and these active sites compete with Cr(VI) and Cu(II) in the solution at the same time, which will be saturated at an optimal concentration. At the same time, the increase in adsorbent dose increases the number of available active sites, which improves the removal efficiency. However, the higher adsorbent dose will also lead to the effect of agglomeration, which reduces the adsorption capacity per unit of adsorbent.

### 3.7 Desirability function (DF)

**Fig. 7:**
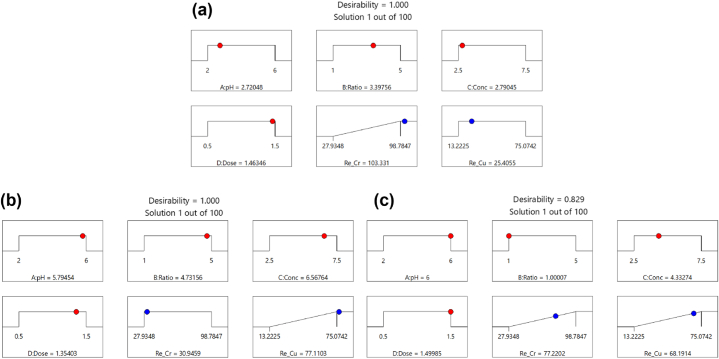
The desirability function for maximum removal efficiency. (a) Cr(VI); (b) Cu(II); (c) both Cr(VI) and Cu(II).

**Fig. 8:**
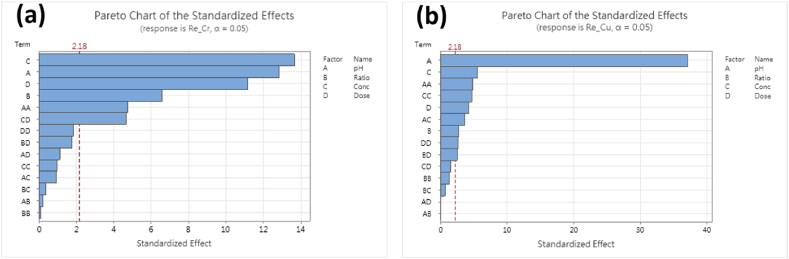
Pareto chart of the standardized effects. (a) Removal of Cr(VI); (b) removal of Cu(II).

The response values of each factor were weighted and combined using a Desirability function (DF) to establish the best prediction conditions to maximize Cr(VI) and Cu(II) removal, with DF ranging from 0 (desirable) to 1 (undesirable). The results are shown in Fig. 7. At the initial pH value of 2.72, M@A (3:3.4), Cr(VI) concentration of 2.79 mg/L, and adsorbent dose of 1.46 g/L obtain maximum Cr(VI) removal efficiency (103.33 %); initial pH value of 5.79, M@A (3:4.73), Cr(VI) concentration of 6.57 mg/L, adsorbent dose of 1.35 g/L, obtain maximum Cu(II) removal efficiency (77.11 %); initial pH value of 6, M@A (3:1), Cr(VI) concentration of 4.33 mg/L, and the adsorbent dose of 1.5 g/L, the simultaneous maximum removal efficiency of Cr(VI) and Cu(II) were 77.22 % and 68.19 %, respectively (see Fig. 8).

**Fig. 9:**
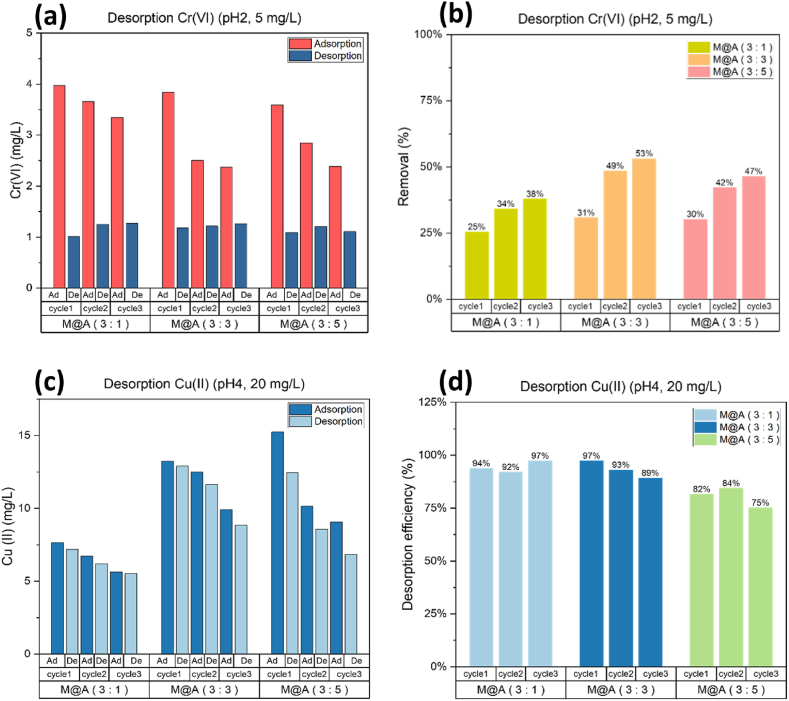
Each cycle of adsorption-desorption amount and efficiency.

To verify the removal efficiency of anionic Cr(VI) and cationic Cu(II) under the best-predicted conditions, the experimental results were compared with the predicted values. The experimental results of the removal efficiency of Cr(VI) and Cu(II) were 82.4 % and 74.0 %, respectively. The verification shows that the agreement between the experimental results and their predicted values means that DF effectively identifies the best-predicted conditions for the simultaneous removal of Cr(VI) and Cu(II) by M@A.

### 3.8 Multiple regression analysis and ANOVA

Table 3 presented the fitting data by multiple regression models, e.g., linear, two-factor interaction, quadratic, and cubic models. The analysis result includes the adjusted R^2^, predicted R^2^, and probability P-value. If the regression model's R^2^ value is closest to 1, that reflects the strength of the model and the best predictability of the response. Finally, the quadratic model has given the best fitness for simultaneous removing Cr(VI) and Cu(II) by adsorption using M@A. The predicted-R^2^ value is 0.8884 and 0.9563 of Cr(VI) and Cu(II), respectively. The final model is presented in Eq to predict the removal percentage values (%) of Cr(VI) and Cu(II).

**Table 3:** Regression model summary.

	Source	Sequential p-value	Lack of Fit p-value	Adjusted R^2^	Predicted R^2^	
Cr(VI)	Linear	<0.0001	0.0799	0.8436	0.7922	
	2FI	0.2669	0.0856	0.8597	0.715	
	**Quadratic**	**0.0009**	**0.2453**	**0.9566**	**0.8884**	**Suggested**
	Cubic	0.2887	0.2574	0.9723	0.5419	Aliased
Cu(II)	Linear	<0.0001	0.0364	0.9138	0.8857	
	2FI	0.6668	0.0321	0.9056	0.8088	
	**Quadratic**	**< 0.0001**	**0.1624**	**0.9832**	**0.9563**	**Suggested**
	Cubic	0.2327	0.1881	0.9907	0.8314	Aliased

ANOVA evaluates the effective parameters in the quadratic regression model, and the results are shown in Table 4. For removal of Cr(VI), coefficient of variation (C.V.) is 7.66 %, standard deviation (Std. Dev.) is 4.44, quadratic regression model p-value <0.0001, and lack of fit p-value is 0.2453. For Cu(II) removal: coefficient of variation (C.V.) is 5.55 %, standard deviation (Std. Dev.) is 2.29, p-value of quadratic regression model <0.0001, and lack of fit p-value is 0.1624. The above analysis confirms the high accuracy of this model [36].

**Table 4:** ANOVA and fit statistics summary.

Cr(VI) Removal						
ANOVA for Quadratic Model						
**Source**	**Sum of Squares**	**df**	**Mean Square**	**F-value**	**p-value**	
**Model**	**11547.9**	**14**	**824.85**	**41.91**	**< 0.0001**	**significant**
A-pH	3241.85	1	3241.85	164.73	<0.0001	
B-Ratio	864.71	1	854.71	43.43	<0.0001	
C-Cone	3672.99	1	3672.99	186.64	<0.0001	
D-Dose	2445.21	1	2455.21	124.76	<0.0001	
AB	0.8713	1	0.8713	0.0443	0.8369	
AC	17.25	1	17.25	0.8764	0.3677	
AD	24.73	1	24.73	1.26	0.2842	
BC	3.04	1	3.04	0.1547	0.701	
BD	62.84	1	62.84	3.19	0.0992	
CD	433.27	1	433.27	22.02	0.0005	
A^2^	447.78	1	447.78	22.75	0.0005	
B^2^	0.2244	1	0.2244	0.0114	0.9167	
C^2^	18.41	1	18.41	0.9356	0.3525	
D^2^	68.14	1	68.14	3.46	0.0874	
Residual	236.15	12	19.68			
**Lack of Fit**	**223.23**	**10**	**22.32**	**3.45**	**0.2453**	**no significant**
Pure Error	12.92	2	6.46			
Cor Total	11784.1	26				
Fit Statistics						
Std. Dev.	4.44	R^2^	0.98			
Mean	57.94	Adjusted R^2^	0.9566			
C.V. %	7.66	Predicted R^2^	0.8884			
		Adeq Precision	21.2117			

Cu(II) Removal						
ANOVA for Quadratic Model						
**Source**	**Sum of Squares**	**df**	**Mean Square**	**F-value**	**p-value**	
**Model**	**8056.91**	**14**	**575.49**	**109.64**	**< 0.0001**	**significant**
A-pH	7230.14	1	7230.14	1377.45	<0.0001	
B-Ratio	40.36	1	40.36	7.69	0.0169	
C-Cone	162.3	1	162.3	31.09	0.0001	
D-Dose	93.9	1	93.9	17.89	0.0012	
AB	0	1	0	5.47E-06	0.9982	
AC	72.19	1	72.19	13.75	0.03	
AD	0.0006	1	0.0006	0.0001	0.917	
BC	2.7	1	2.7	0.5147	0.4868	
BD	33.44	1	33.44	6.37	0.0267	
CD	12.29	1	12.29	2.34	0.1519	
A^2^	123.23	1	123.23	23.48	0.0004	
B^2^	10.17	1	10.17	1.94	0.1891	
C^2^	116.86	1	116.86	22.26	0.0005	
D^2^	37.61	1	37.61	7.17	0.0202	
Residual	62.99	12	5.25			
**Lack of Fit**	**60.79**	**10**	**6.08**	**5.54**	**0.1624**	**no significant**
Pure Error	2.19	2	1.1			
Cor Total	8119.9	26				
Fit Statistics						
Std. Dev.	2.29	R^2^	0.9922			
Mean	41.27	Adjusted R^2^	0.9832			
C.V. %	5.55	Predicted R^2^	0.9563			
		Adeq Precision	35.0179			

Pareto Chart of the standardized effects analyzes the magnitude and importance of each factor on the response, as shown in Fig. 8. On the removal of Cr(VI), it is shown that the four main factors all have a significant effect on the removal results, and their magnitudes are in the order of Cr(VI) concentration > initial pH > adsorbent dose > ratio of M@A. On the removal of Cu(II), it shows that the initial pH value has a great influence, and the rest are in the order of Cr(VI) concentration > adsorbent dose > ratio of M@A.

### 3.9 Adsorption-desorption cycle

In the adsorption-desorption cycle of Cr(VI), the desorption environment was added to an equal volume of 0.5 M NaOH + 2.5 M NaCl and stirred continuously at room temperature for 90 min. The adsorption and desorption amounts and desorption efficiency in each cycle are shown in Fig. 9a and b. It can be found that the adsorption capacity of the three adsorbents gradually decreases with the increase of the cycle number, but the desorption capacity remains constant at about 1.2 mg/L. Similarly, for Cu(II) desorption added 0.2 M HCl and stirred for 30 min. The adsorption and desorption amounts and desorption efficiency in each cycle are shown in Fig. 9c and d, 0.2M HCl can desorb almost all the Cu(II), and the three adsorbents can still maintain their effectiveness after three cycles.

## 4 Conclusion

Through comprehensive experimental and analytical data, this study successfully synthesized and characterized MNP composites with microalgae biomass. The TGA, FTIR) and SQUID measurements confirmed the correct properties and successful synthesis of the M@A composites. TGA analysis demonstrated a mass loss rate of 94.93 % for MNPs, indicating very high thermal stability. FTIR analysis revealed characteristic peaks of Fe_3_O_4_ nanoparticles, verifying the presence of magnetic nanoparticles and their successful integration with microalgae. SQUID measurements confirmed the magnetic properties of the composites, supporting their successful synthesis and performance evaluation.

The M@A composites exhibited significant adsorption capacity for the simultaneous removal of Cr(VI) and Cu(II) ions from aqueous solutions. Utilizing the BBD, experimental parameters were optimized, including initial pH, M@A ratio, Cr(VI) concentration, and adsorbent dose. Under optimal conditions (pH 6, Cr(VI) concentration of 5 mg/L, Cu(II) concentration of 20 mg/L, and M@A (3:3) dose of 1 g/L), the removal efficiencies for Cr(VI) and Cu(II) were 58 % and 73.4 %, respectively.

A quadratic regression model was developed to predict the maximum removal efficiencies of Cr(VI) and Cu(II) using M@A composites. The model exhibited high predictability, with adjusted R^2^ values of 0.9566 and 0.9832 for Cr(VI) and Cu(II) removal, respectively. This model effectively identifies optimal adsorption conditions, providing scientific validation for industrial applications.

The M@A composites showed excellent reusability, maintaining good adsorption capacity over multiple adsorption-desorption cycles. The desorption process using NaOH and HCl effectively restored the adsorption sites, ensuring that the composites could be reused for at least three cycles without significant loss of efficiency. This demonstrates the potential of M@A composites for practical wastewater treatment.

The use of M@A composites offers significant environmental and economic advantages. These materials are eco-friendly, cost-effective, and can be rapidly separated from the solution using a magnetic field, reducing the time and cost associated with traditional separation processes. This makes M@A a promising candidate for large-scale applications in removing toxic heavy metals from wastewater.

In summary, the M@A composites developed in this study provide an efficient and sustainable solution for removing Cr(VI) and Cu(II) ions from aqueous solutions. The successful integration of microalgae and magnetic nanoparticles not only enhances adsorption capacity but also simplifies recovery and reuse processes. This research offers a new perspective and methodology for environmental remediation technologies and lays a solid scientific foundation for future practical applications in wastewater treatment.

## CRediT authorship contribution statement

**Huan-Cheng Lin:** Writing – original draft, Data curation, Conceptualization. **Yi-Ju Liu:** Writing – review & editing, Methodology, Investigation, Conceptualization. **Da-Jeng Yao:** Writing – review & editing, Project administration, Conceptualization.

## Declaration of competing interest

The authors declare that they have no known competing financial interests or personal relationships that could have appeared to influence the work reported in this paper.

Da-Jeng Yao reports was provided by 10.13039/501100005057National Tsing Hua University. Da-Jeng Yao reports financial support was provided by 10.13039/100020595National Science and Technology Council. Da-Jeng Yao reports a relationship with 10.13039/501100005057National Tsing Hua University that includes: employment. If there are other authors, they declare that they have no known competing financial interests or personal relationships that could have appeared to influence the work reported in this paper.
